# Statistical power considerations in genotype-based recall randomized controlled trials

**DOI:** 10.1038/srep37307

**Published:** 2016-11-25

**Authors:** Naeimeh Atabaki-Pasdar, Mattias Ohlsson, Dmitry Shungin, Azra Kurbasic, Erik Ingelsson, Ewan R. Pearson, Ashfaq Ali, Paul W. Franks

**Affiliations:** 1 Department of Clinical Sciences, Genetic and Molecular Epidemiology Unit, Lund University, Malmö, SE-205 02, Sweden; 2 Department of Astronomy and Theoretical Physics, Computational Biology and Biological Physics Unit, Lund University, Lund, Sweden; 3 Department of Medical Sciences, Molecular Epidemiology, Uppsala University, Uppsala, Sweden; 4 Division of Cardiovascular & Diabetes Medicine, Medical Research Institute, University of Dundee, Dundee, UK; 5 Department of Public Health & Clinical Medicine, Umeå University, Umeå, Sweden; 6 Department of Nutrition, Harvard School of Public Health, Boston, MA, USA

## Abstract

Randomized controlled trials (RCT) are often underpowered for validating gene-treatment interactions. Using published data from the *Diabetes Prevention Program* (DPP), we examined power in conventional and genotype-based recall (GBR) trials. We calculated sample size and statistical power for gene-metformin interactions (vs. placebo) using incidence rates, gene-drug interaction effect estimates and allele frequencies reported in the DPP for the rs8065082 *SLC47A1* variant, a metformin transported encoding locus. We then calculated statistical power for interactions between genetic risk scores (GRS), metformin treatment and intensive lifestyle intervention (ILI) given a range of sampling frames, clinical trial sample sizes, interaction effect estimates, and allele frequencies; outcomes were type 2 diabetes incidence (time-to-event) and change in small LDL particles (continuous outcome). Thereafter, we compared two recruitment frameworks: GBR (participants recruited from the extremes of a GRS distribution) and conventional sampling (participants recruited without explicit emphasis on genetic characteristics). We further examined the influence of outcome measurement error on statistical power. Under most simulated scenarios, GBR trials have substantially higher power to observe gene-drug and gene-lifestyle interactions than same-sized conventional RCTs. GBR trials are becoming popular for validation of gene-treatment interactions; our analyses illustrate the strengths and weaknesses of this design.

Genome-wide association studies (GWAS) have identified thousands of loci associated with complex traits^1^,^2^,^3^,^4^,^5^,^6^,^7^,^8^, the clinical relevance of which is sometimes assessed in existing randomized controlled trials (RCT) by testing for effect-modification by modeling gene-treatment (e.g., drugs or lifestyle) interactions^9^,^10^.

Data on gene-treatment interactions may help optimize therapy, as determining the nature and extent of interactions can elucidate mechanisms and illustrate differences in treatment efficacy by genetically distinguishable subpopulations^10^, a concept broadly related to *precision medicine*. This is relevant when the treatment’s efficacy is heterogeneous, with some patients who respond well and others who do not, and when treatments are expensive or convey serious side effects in a patient subgroup.

Modeling gene-treatment interactions in existing Phase III RCTs is a pragmatic use of existing data; however, most trials were not designed for this purpose, often lack power, and may not focus on the most relevant phenotypes or treatments. Thus, genotype based recall (GBR) trials, which are designed explicitly to test given hypotheses about gene-treatment interactions, represent an alternative approach that is gaining traction in academia and industry, as focusing on genetically at-risk participants might enhance power to assess treatment efficacy by virtue of accelerated outcome incidence rates and/or enhanced drug sensitivity^11^,^12^,^13^.

Statistical power to observe gene-treatment interactions is conditional on several factors, not least the effect allele frequency; thus, increasing the number of effect allele carriers within a clinical trial population should enhance power. This can be achieved in RCT cohorts by recruiting participants with specific genotypes (GBR)^14^. For complex traits, gene-lifestyle or gene-drug interactions for a single locus tend to be small in magnitude; therefore, the amalgamation of multiple variants into genetic risk scores (GRS) might enhance power. When the objective is to test interactions using a GRS, GBR trials focus on two distinct subgroups, one with many and the other with few effect alleles at the loci of interest, with the size of the initial sampling-frame determining the extent to which genetic risk can be juxtaposed. When a single rare variant is the focus, the GBR approach can be used to equilibrate risk allele frequencies, potentially increasing power.

The purpose of this simulation study was to compare power and sample-size requirements to observe gene-treatment interactions using the GBR and conventional recruitment paradigms for RCTs. To maximize the relevance of this work, we compared results from GBR trial simulations with those for conventional trials using published data from the Diabetes Prevention Program (DPP), an existing Phase III RCT of drug and lifestyle interventions. The results of these analyses are presented in web-based power/sample size calculator, which we anticipate will facilitate the design of future GBR trials (https://gbr-power.crc.med.lu.se).

## Materials and Methods

### Diabetes Prevention Program (DPP)

To inform the assumptions that underlie the statistical power models described below, we drew on published data from the DPP trial. Initially ~31,000 people were screened with an oral glucose tolerance test (OGTT)^15^. Of these, 3,234 overweight/obese adults from five ethnic groups with elevated fasting and 2-hr post-load plasma glucose concentrations were randomized to receive metformin (850 mg twice daily, n = 1,073), or intensive lifestyle intervention (ILI) (n = 1,079), focusing on weight loss through exercise (150 min moderate-to-high intensity activity per week) and diet; the placebo control arm received sham metformin pills and standard-of-care (n = 1,082)^16^. The primary outcome was diabetes incidence confirmed by repeated fasting or 2-hr plasma glucose concentrations (≥7.0 or ≥11.1 mmol/l respectively), obtained at semi-annual screening visits. At baseline and/or 1 yr post-randomization, biomarkers, including lipoprotein sub-fractions and genotypes (n = 2,994 participants consented to genotyping)^9^ were assayed (see ref. 17 for further details).

### Genetic variables, gene-treatment interactions and population scenarios

We used parameters for two phenotypes reported by the DPP focused on: i) incidence of type 2 diabetes (T2D) in the metformin and ILI arms for interaction metrics between a variant at the *solute carrier family 47 multidrug and toxin extrusion*, *member 1* (*SLC47A1*) locus and metformin (vs. placebo) in relation to diabetes incidence^9^; ii) interaction metrics between a GRS (32 SNPs) and ILI (vs. placebo) in relation to 1-yr changes in small LDL particle size^10^. Data on genetic effects and gene-treatment interaction effects were obtained from these papers and used in the following models. First we calculated sample sizes and statistical power to detect interactions according to the DPP parameters for the conventional RCT and GBR settings.

To generate additional statistical power data that extends the DPP examples described above, we calculated statistical power for genetic interactions with metformin or ILI interventions in relation to disease incidence and change in a quantitative trait focusing on a GRS comprised of 20 SNPs (an arbitrary number) with moderate-high minor allele frequencies (MAF), small or large effects, and varying degrees of outcome measurement error.

For the DPP-specific examples, we used diabetes incidence and follow-up duration and the per allele interaction effect estimate (HR = 0.68) between a variant at the *SLC47A1* locus (MAF = 0.44) and metformin (vs. placebo), as reported in the DPP^16^. We adopted a sampling-frame of 31,000, consistent with the DPP’s sampling-frame^17^. For GRS × ILI interactions, we calculated an unweighted GRS by simulating 32 SNPs with risk allele frequencies reported in the DPP (n = 1,909)^10^ and by summing risk alleles, as previously described^18^. Summary statistics obtained from this GRS were then used to simulate the GRS variable in the sampling-frame. The per allele interaction effect reported in the DPP for change in small LDL particles is 0.03 nmol/l given lifestyle intervention, and is used in our simulations.

We then assessed interactions between treatments (metformin or ILI vs. placebo) and SNPs with moderate (MAF = 0.05–0.2) and high (MAF = 0.2–0.5) frequencies, with small (β = 0.05–0.15) and large (β = 0.15–0.30) effects, individually (SNPs) and together (GRSs). “Small effects” correspond to per allele diabetes HRs of 0.95–0.86, while “large effects” correspond to HRs of 0.85–0.74 in the metformin arm (vs. placebo). Small and large effect sizes correspond to 0.05 to 0.15 nmol/l/yr decrease and 0.15 to 0.30 nmol/l/yr decrease in small LDL particles, respectively, per risk allele with ILI (vs. placebo)^10^. The GRSs were calculated assuming the genotype distributions conformed to Hardy-Weinberg expectations. The maximum theoretical range of the GRS was 0–40 risk alleles, and the GRS was normally distributed. To confirm this assumption of normality, we modeled 20 random, uncorrelated SNPs from the GLACIER cohort (n = 6,064)^19^ with MAFs of 0.20–0.50 (Supplementary Figure 1).

When simulating a GBR trial, we sampled directly from the most extreme point of each tail and worked inwards towards the centre point. Therefore, no explicit cut-point was used.

Marginal and interaction effects for diabetes incidence were simulated using Cox proportional hazards models, and linear regression for 1-yr change in small LDL particles, with treatment, GRS, and GRS × treatment interactions fitted as independent variables.

In addition to considering different allele frequencies and effect estimates, we also considered the impact that the size of the sampling-frame (N = 5,000, 10,000, 50,000, 100,000 and 500,000) and measurement error have on power. To assess the impact of measurement error, we generate outcomes reflecting those assessed with different levels of precision, described by the correlation (r^2^) between a criterion measure and weaker measure of the outcome (r^2^ of 0.8, 0.6 and 0.4 representing high, moderate and low precision respectively).

We provide an online power calculator (https://gbr-power.crc.med.lu.se), through which power or sample size can be calculated for other scenarios (more/less SNPs, lower MAFs, and different outcomes).

### Simulations for GRS × metformin interactions and calculation of summary statistics for diabetes incidence

To calculate the underlying survival time variable we used the method described by Bender *et al*.^20^. The survival time as a function of predictor variables and their effect is estimated using the following formula (equation (1)) and is defined as the time-to-event for each participant given the covariates such as metformin and SNPs:


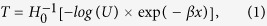


where *T* denotes the survival time, *U* follows a uniform distribution on the interval [0, 1], *x* is a matrix of covariates, 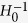 denotes the inverse cumulative baseline hazard function when *x* is zero and *β* is a vector of regression coefficients for marginal effects of all the SNPs and their interactions with metformin. The matrix *x* contains vectors for covariates such as metformin, individual SNP variables and variables of the cross product of the SNP and metformin. The longest follow-up time was set to 4.0 years and overall type 2 diabetes incidence was set to 37% at the study endpoint as approximated from published DPP data^16^. To obtain summary statistics for Cox-proportional hazard models, the model defined in equation (2) was fitted to data using the *coxph* function in the R program^21^, where the GRS variable was used as a covariate in the regression model.


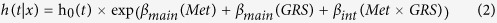


### Simulations for GRS × ILI interactions and 1-yr change in small LDL particle size

We calculated 1-year small LDL particle size using the equation summarized in equation (3).


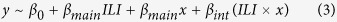


where *β*_*main*_ is the vector of marginal effects for the 20 index SNPs; *β*_*int*_ is the vector of interaction effects; and *x* is the matrix of SNP vectors with corresponding allele frequencies; and y is the estimated small LDL particle size for each individual based on population parameters specified in equation (3) according to the estimates published by the DPP consortium or as mentioned above.

### Models for effect size estimates

To obtain summary statistics for linear models, the following model was used to calculate summary statistics using the *lm* function in the R program^21^, where the GRS variable was used as a covariate in the regression model (equation (4)).

In order to obtain summary statistics for marginal and interaction effects, the following regression equation was modeled:


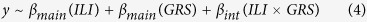


### Statistical power calculation

We implemented simulations to provide an estimate of power likely to result from subsequent RCTs given various sample sizes. To determine the number of iterations needed to derive precise estimates of power, we tested various iterations from 10–2,000 at frequent integrals. We found that in almost all scenarios ≤1,000 iterations were needed (often only 100) to achieve reliable power estimates; however, for the sake of consistency, 1,000 iterations are used in all of the power calculations included in the web-based power calculator.

We used two methods to calculate statistical power for interaction effects:

The *Zero*/*One* method was used to calculate power from the Cox simulations. Each simulation was scored with 1 when the *P*-value for the interaction β, taken from the summary of the *coxph* function, was <0.05, and 0 for the simulations where the *P*-value for the interaction β was ≥0.05. We then summed the number of scores equal to one and divide by the total number of simulations to estimate power.

For linear models, the *standard error* method was used. We took the effect size and standard error of the interaction term from the summary of the lm function in R for each simulation, and then averaged these effect sizes and standard errors respectively, and incorporated these into equation (5):


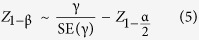


where γ is the effect size (i.e. the difference between the value of null hypothesis and alternative hypothesis for the parameter being tested) SE (γ) is the standard error of the effect size (a function of sample size), α is the significance level, 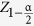 is the z-score of 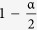, β is the probability of making a type 2 error and *Z*_1−β_ is the z-score of 1 − β and representing the power of the test^22^.

Applying the *Zero*/*One* method to calculate power for interaction effects modeled with linear regression yielded the same results as the standard error method.

When using the Standard Error method (equation 5 above), α is the significance level in the equation, which denotes the probability of type 1 error. Applying the Zero/One method, we also consider type 1 error by scoring each simulation with 1 when the *P*-value for the parameter of interest is below the significance level (here 0.05), and 0 where the *P*-value is equal to or above 0.05. An entry field labeled as “Significance Level” is also provided in the web interface for the type 1 error rate the user specifies. In all simulations described here, type 1 error rates were set to 0.05 for both study designs to avoid bias.

## Results

### Statistical power to observe the interaction between the rs8065082 variant at *SLC47A1* and metformin treatment (vs. placebo control) in diabetes incidence, as reported in the DPP

**Figure 1:**
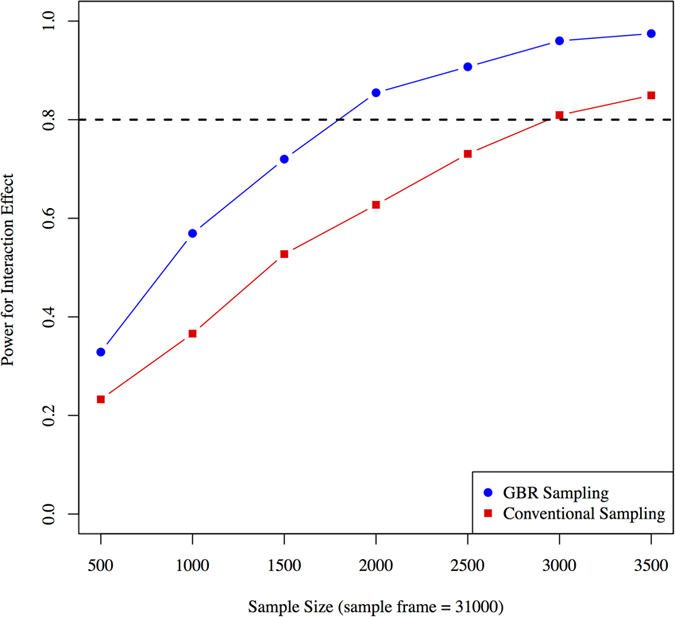
Statistical power (y axis) to observe an interaction between variant rs8065082 at *SLC47A1* and metformin using GBR and conventional sampling approaches (based on DPP clinical trial parameters).

With a sampling-frame of 31,000 individuals, power to observe an interaction between the rs8065082 variant and metformin was higher with the GBR approach compared with the conventional sampling approach. For example, a sample size of ~3,000 is required to achieve 80% power using conventional sampling, while 80% power can be achieved with ~1,700 participants using the GBR approach (Fig. 1).

### Impact of MAFs and effect sizes on GRS × treatment interactions in diabetes

**Figure 2:**
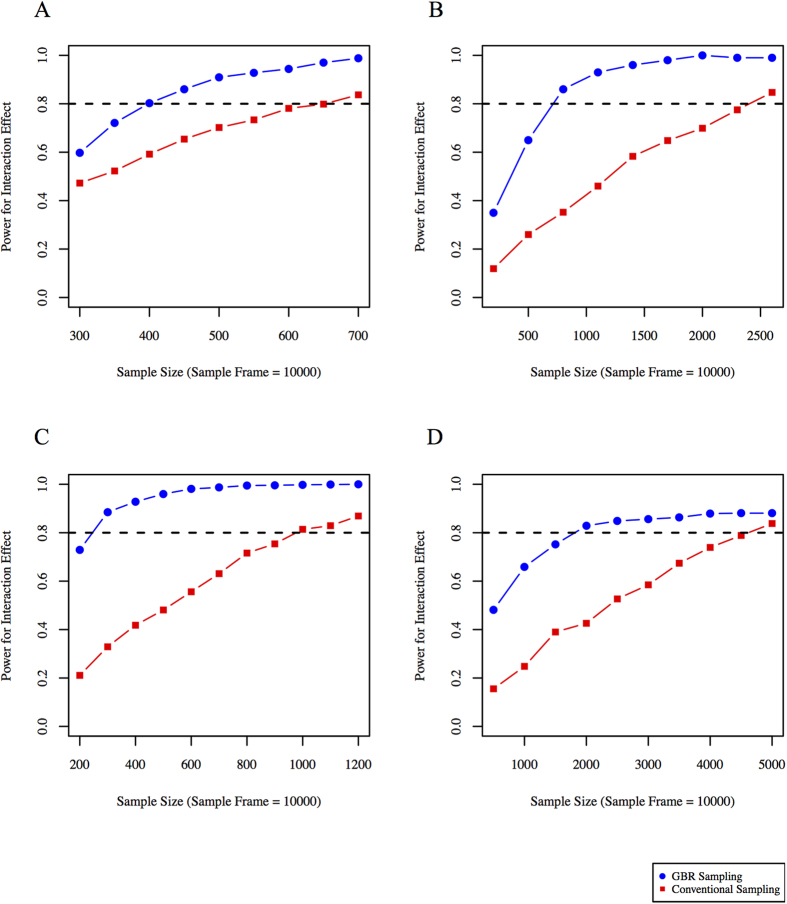
Statistical power (y axis) for different sample sizes using conventional sampling and GBR approach: (**A**) high-frequency SNPs with large effect sizes, (**B**) high-frequency SNPs with small effect sizes, (**C**) moderate-frequency SNPs with large effect sizes, and (**D**) moderate-frequency SNPs with small effect sizes. Simulations performed using Cox-proportional hazards models for time to event data.

With a sampling-frame of 10,000 individuals, statistical power to observe GRS × treatment interactions was significantly higher for the GBR approach compared with the conventional sampling approach in all four tested scenarios; these scenarios are: high-frequency SNPs with large effect size; high-frequency SNPs with small effect size; moderate-frequency SNPs with large effect size; and moderate-frequency SNPs with small effect size. For high-frequency SNPs with large effect sizes, trials with ~390 and ~650 participants would be required to achieve 80% power to observe a GRS × metformin interaction using the GBR and conventional sampling approaches, respectively (Fig. 2A). For high-frequency SNPs with small effect sizes, these sample sizes are ~700 and ~2,500 respectively (Fig. 2B). For moderate-frequency SNPs with large effect sizes, these sample sizes are ~250 and ~1,000 respectively (Fig. 2C). For moderate-frequency SNPs with small effect sizes, these sample sizes are ~1,700 and ~4,500 respectively (Fig. 2D).

### Statistical power to observe the GRS × ILI interaction for 1-yr change in small LDL particles reported in the DPP

**Figure 3:**
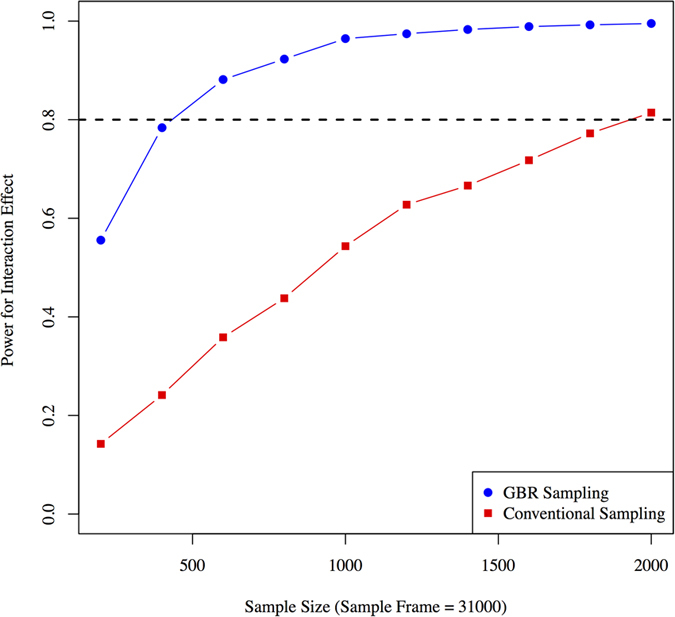
Statistical power to observe GRS × lifestyle interactions on 1-year small LDL particle levels (based on DPP clinical trial parameters).

Taking a sampling-frame of 31,000 and a 32 SNP GRS, the GBR approach yields higher statistical power than the conventional sampling approach for different sample sizes (Fig. 3). To obtain 80% power with the given assumptions, ~400 participants are required using the GBR approach, whereas ~1,900 would be required with conventional recruitment.

### Impact of MAFs and effect sizes on GRS × treatment interactions for 1-yr change in small LDL particles

**Figure 4:**
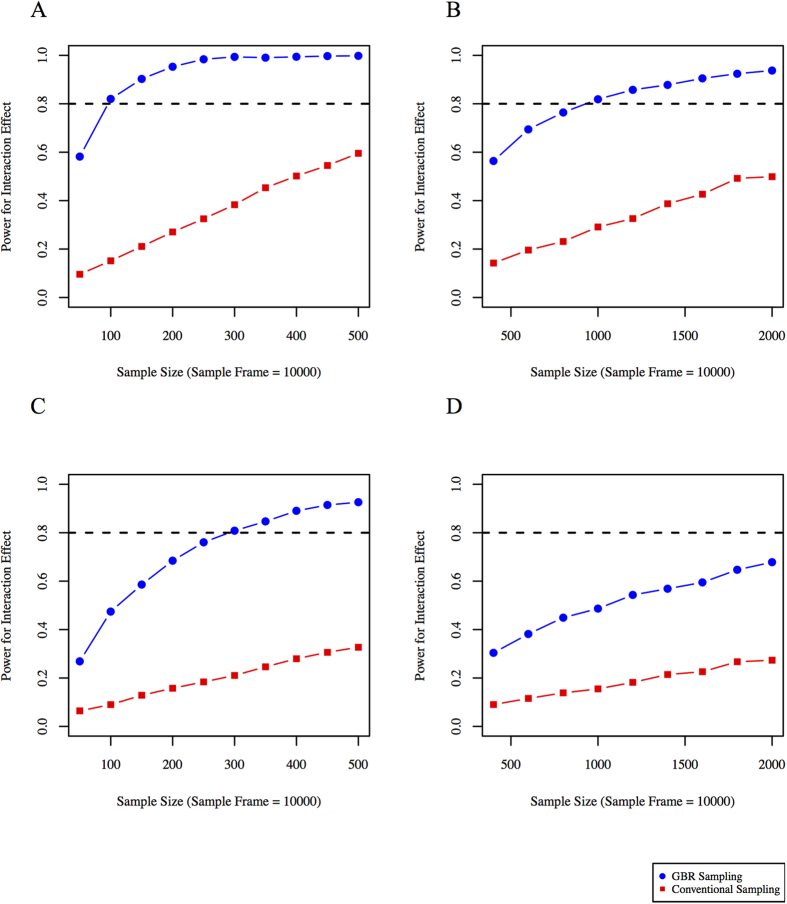
Statistical power (y axis) for different sample sizes using conventional sampling and GBR approaches for trials design to observe gene × treatment (lifestyle vs. control) interactions for 1-year small LDL particle levels. Genetic risk score with different underlying allele frequencies and effect sizes are considered: (**A**) high-frequency SNPs with large effect sizes, (**B**) high-frequency SNPs with small effect sizes, (**C**) moderate-frequency SNPs with large effect sizes, and (**D**) moderate-frequency SNPs with small effect sizes. Simulations were performed using linear regression.

Like the examples given above using Cox proportional hazards models, the GBR approach yields significantly higher power compared with the conventional sampling approach in all tested scenarios (high-frequency SNPs with large and small effect sizes and moderate-frequency SNPs with large and small effect sizes). With a sampling-frame of 10,000 participants, ~100 and ~700 participants would be required using the GBR and the conventional sampling approaches respectively to observe a GRS × ILI interaction where the GRS is comprised of high-frequency SNPs with large effects (Fig. 4A). With a GRS comprised of high-frequency SNPs conveying small effects, the sample sizes required to achieve 80% power are ~1,000 and ~3,900 respectively (Fig. 4B). With a GRS comprised of moderate-frequency SNPs that convey large effects, these sample sizes are ~285 and ~1,600 respectively (Fig. 4C). With a GRS comprised of moderate-frequency SNPs with small effect sizes, these sample sizes are ~2,700 and ~7,000 respectively (Fig. 4D).

### Effect of sampling-frame size on statistical power in GRS × treatment interactions

**Figure 5:**
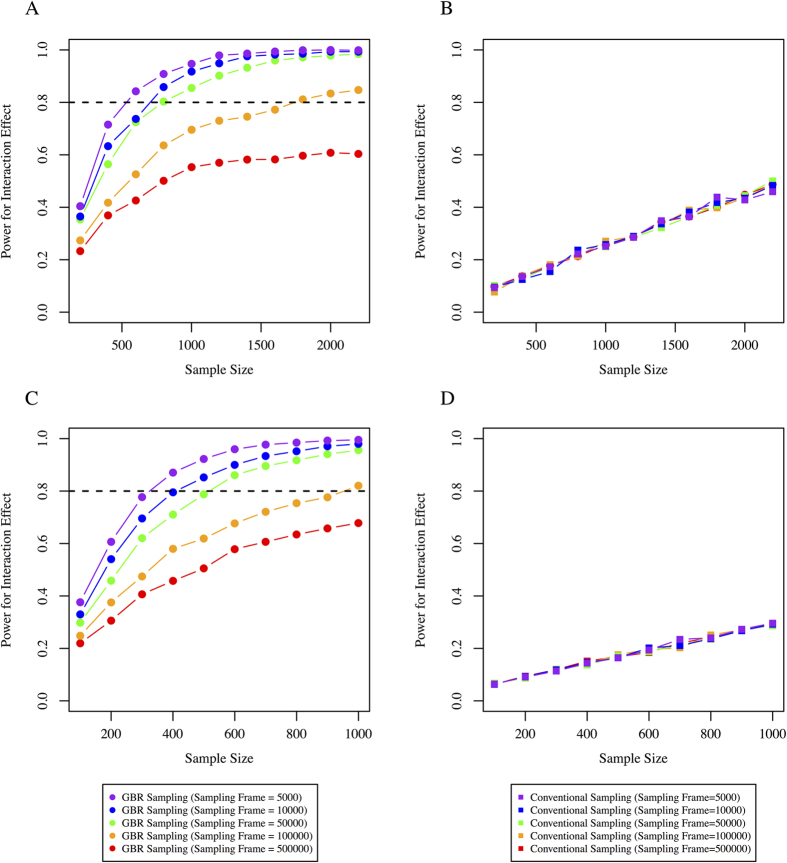
Comparison of the conventional sampling approach with the GBR approach. Statistical power is shown on the y axis and sample size on the x axis from a range of initial sampling-frames. GRS based on high-frequency SNPs with small effect estimates were considered and simulations performed for: (**A**) Cox proportional hazard model for time to event data for GBR sampling, (**B**) Cox proportional hazard model for time to event data for conventional sampling, (**C**) Linear regression models for 1-year small LDL particle levels for GBR sampling, and (**D**) Linear regression models for 1-year small LDL particle levels for conventional sampling.

**Figure 6:**
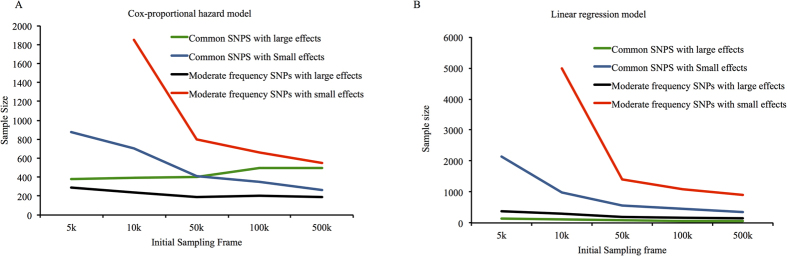
Sample size (y axis) required for 80% power for different initial sampling-frames (x axis) and different allele frequencies and effect estimates. Line color indicates different scenarios for genetic variant allele frequencies and effect estimates: (**A**) Cox-proportional hazard model, (**B**) linear regression model.

Changing the sampling-frame size does not materially affect the mean of the GRS, but the number of observations at the extremes of the GRS distribution grows as the sampling-frame increases in size (Supplementary Figure 2). This has no material impact on statistical power when participants are randomly sampled from the population, as in conventional clinical trials. However, for the GBR approach, increasing the sampling-frame yields a progressively greater genetic contrast between the two recalled groups, thereby increasing power (Fig. 5A–B for Cox and 5C-D for linear models) in almost all scenarios. For example, where high-frequency variants with small effects were considered, increasing the initial sampling-frame (5,000 through 10,000, 50,000, 100,000, 500,000 participants) significantly improves statistical power (Fig. 5A–B). Similarly, for the linear model, the GBR approach yielded significantly higher power. Increasing the initial sampling-frame had no effect on statistical power in the conventional trial scenario. Figure 6 shows comparisons for a range of other scenarios, each of which demonstrates higher power to observe interactions for the GBR vs. conventional sampling approaches.

### Measurement error and power to observe interactions

**Figure 7:**
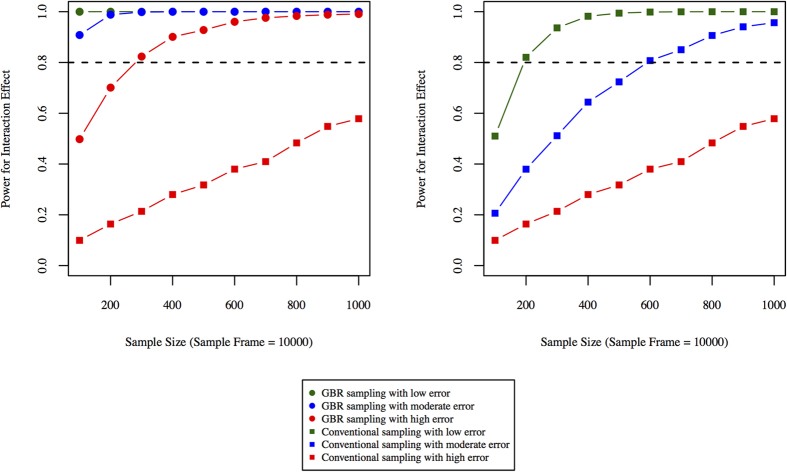
Effect of measurement error on power: (**A**) shows power curves for phenotypes (outcomes) measure with varying degree of measurement error using the GBR paradigm, (**B**) shows power with different sample sizes using the conventional participant recruitment paradigm. Statistical power is plotted on the y-axis and samples sizes are plotted on x-axis.

The smaller sample size requirements of GBR trials might permit deeper or more precise phenotyping of participants. This is perhaps most relevant when statistical power to observe interaction effects remains low, even when the GBR approach is used. Because error in the assessments of exposures and outcomes profoundly influences power to observe interaction effects^23^, we proceeded to examine the extent to which reductions in measurement error influence sample size requirements and power. As Fig. 7 shows, power to observe interactions improves in both settings, but there are scenarios where power is suboptimal in conventional trials even when the outcomes are precisely assessed, but where improvements in measurement precision in a GBR trial raises power to an acceptable level. The required sample sizes to reach 80% power using the GBR approach were ∼20, ∼70 and ∼300 for low, moderate and high error, respectively. With conventional sampling, the required sample sizes were ∼200, ∼600 and ∼1,800 for low, moderate and high error, respectively.

## Discussion

We assessed plausible scenarios within which one might consider testing genotype × treatment (drug or lifestyle) interactions. Using the population parameters and effect estimates from the DPP for time-to-event^9^ and quantitative^10^ outcome variables, we demonstrate through simulations that the GBR approach is ubiquitously more powerful than the conventional sampling approach used in clinical trials when the objective is to validate a genotype × treatment interaction.

To place our data in context with real-world examples of genotype × treatment interactions, we undertook simulations using published data from the DPP, an RCT within which genotype × treatment interactions have been reported for metformin^9^ and lifestyle^10^ interventions. Our simulations suggest that with a total of 2,155 participants randomized to the metformin and placebo control interventions, the DPP was likely underpowered (61%) to observe the interaction between the rs8065082 *SLC47A1* variant and metformin in type 2 diabetes incidence. A trial of equivalent design to the DPP would require roughly 3,000 participants to achieve 80% power to observe this interaction effect; a comparable level of power could be achieved in a GBR trial comprised of as few as 1,700 participants (Fig. 1).

We show that recruiting clinical trial participants based on predetermined genetic characteristics is often a substantially more powerful approach for validating gene × treatment interaction effects than conventional approaches, where participants are recruited randomly and subsequently genotyped. Nevertheless, there are scenarios where GBR trials are likely to be especially appealing; for example, where the genotype of interest is infrequent or where the procedures or assays needed to adequately phenotype participants are very costly or challenging to perform.

In scenarios where the index genotypes are very common within the background population, the GBR approach is less compelling, not least because it has considerable caveats. For example, trials that recruit participants by genotype are by definition designed in such a way that the trial population is genetically unrepresentative of the background population. Thus, unlike conventional RCTs, GBR trials are unlikely to be suitable for extensive secondary hypothesis testing. Furthermore, GBR trials of the nature described here are unlikely to be suitable for discovery of genetic loci that interact with treatment, owing to the special genetic characteristics of the trial cohort and the specific hypothesis GBR trials are designed to test, whereas it is conceivable that very large conventional RCTs might be suitable for this purpose. Nevertheless, focusing recruitment on the subgroup of the population at the highest genetic risk of the trial’s outcome (rather than adopting the approach we define here of juxtaposing high and low risk participants for a specific locus or set of loci), is likely to improve power for discovery of novel genetic variants that interact with treatments in clinical trials; this, as Shork and Topol eloquently quantified^12^, is primarily attributable to the higher incidence rates of the trial’s outcome anticipated in people at high genetic risk. However, as the authors note, most genetic variants that might be used for this purpose have been discovered using prevalent outcome data, and a core assumption that is made in these analyses, that variants that predispose to higher odds of prevalent disease also raise incidence rates, is yet to be shown for most susceptibility variants. It is also important to keep in mind that if the genotypes upon which participants are recalled influence retention, power to observe interactions may be diminished and results may be biased. Thus, when GBR trials are designed, it would be prudent to first determine whether the genotypes of interest are associated with retention, which could be done using analyses of existing RCT data.

Our work focuses predominantly on scenarios where genotype might influence response to interventions. It is also possible that the same genotypes might affect the residual risk of developing the disease or trait of interest (i.e., the variant conveys both marginal and interaction effects), which would affect power^12^. To address this, the marginal and interaction effect estimates are incorporated into our calculations (and can also be adjusted in the web calculator by the user).

Some of our analyses focus on an intronic variant at *SLC47A1*, which is unlikely to be functional. We elected to do so because we sought a real-world, published comparison^9^. However, it is important to keep in mind when designing GBR trials that the observation of gene-treatment interactions is likely to be greatest when participants are recalled on genotypes at functional loci rather than their non-functional proxies, as the latter will result in a degree of exposure misclassification. The extent to which power will diminish depends on the degree of linkage disequilibrium between the functional and proxy variants, such that low linkage disequilibrium between variants will result in greater losses in power, in much the same way as phenotype assessment error affects power (Fig. 7).

There are also specific ethical considerations for GBR trials. Many new cohort studies request participants to consent to be recalled for subsequent sub-studies, such as GBR trials. In the UK, for example, the UK Biobank (N~500,000)^24^, INTERVAL (N~50,000)^25^, and NIHR BioResource (N~20,000–100,000)^26^ are all designed with future GBR studies in mind. Similar initiatives are underway in other countries. However, where GBR studies are planned in cohorts that began long before the GBR study design was established, it is unlikely that appropriate consent was obtained; in these settings, it may be necessary to re-consent participants before GBR can be initiated, or focus the recall on stored biosamples rather than the physical recall of participants^27^.

For some of our analyses, a sample-frame of 31,000 participants was selected as this represents the number screened for the DPP trial. It is worth keeping in mind though that only ~10% of these people were eventually enrolled into the DPP, as many were ineligible. Thus, a GBR trial designed to test the *SLC47A1* hypothesis would require sampling-frames many times larger than 31,000, if enough participants who are fully eligible are to be identified, which has clear cost implications for GBR trials. However, a larger sampling-frame would allow an even more extreme juxtaposition of the two genotype groups, which would further enhance power in the GBR trial. Whilst the cost implications of needing such large sample-frames are important, once the sampling-frame is in place, such as is the case in the UK cohorts outlined above, this limitation is largely offset.

Although we are unaware of other studies that closely parallel ours, Hu *et al*.^28^ assessed the economic implications of enrolling participants at high-risk of disease based on clinical and genetic characteristics, with the expectation that doing so would maximize event rates. The study also helps illustrate the costs and benefits, as well as statistical power implications of recalling participants by genotypes for studies of risk prediction and prevention. Whilst this approach described by Hu *et al*.^28^ differs in many ways to the GBR design we explore here, the core principle of recruiting participants on the basis of their genetic characteristics to enhance the statistical power of an analysis is the same.

At least two published studies describe statistical power for phenotype-based recall studies^29^,^30^, although not in the context of clinical trials. Sampling from the extremes of phenotypic distributions has proven powerful for the detection of rare variants and for studying disease mechanisms in cross-sectional settings. Indeed, the mechanistic understanding of numerous highly penetrant traits has been greatly enhanced by studies designed in this way, particularly when the phenotype is easily observed such as in congenital obesity^31^ and some cancers^32^. Sampling from the extremes of the distribution for a given phenotype increases power to detect rare variants substantially, with as much as a four-fold reduction in sample size requirement^29^,^30^. However, there are significant caveats to phenotype-based recall studies that genotype-based recall studies do not face. For example, most phenotypes are affected by multiple exposures, whereas germline DNA variants are not; thus, confounding in phenotype-based recall studies may be much more difficult to contain than in the genotype-based recall setting. This is especially important in RCTs, where the point is to provide a high level of causal inference.

In addition to the comparison of power in GBR and conventional trials, we also explored the influence of phenotype measurement error on power under the two recruitment scenarios. Our primary aim was not to determine whether error in the assessment of the outcome impacts power to observe interaction effects, as this has been done before^23^,^33^. Instead, we report these results because they are likely to prove useful for those designing GBR trials. Consider, for example, scenarios where even with GBR recruitment a trial is underpowered to observe interactions using standard phenotyping procedures; in these cases, the costs saved by undertaking the trial in fewer participants could be reinvested in the trial by performing more detailed phenotyping or extending the duration of follow-up.

Our simulations are predicated on several core assumptions, almost all of which are described in detail elsewhere in this paper. An additional assumption we make in the GRS models is that genotype × treatment interaction effects are linear across the distribution of these scores. It is possible, however, that the manner in which genotypes at the extremes of a GRS distribution interact with treatments differ from those in the middle of the GRS distribution; this could be easily addressed by including a small group of participants from the middle of the GRS distribution within a GBR trial.

It is also important to keep in mind that the GBR design will not have superior power over conventional trials when: i) the sampling frame is too small to identify sufficient numbers of minor allele carriers or ii) genetic risk scores are used and the recalled groups are not sufficiently distinct.

In some instances, genetic variables will be strongly correlated with other factors (phenotypes) that can be easily measured, and recruiting on these variables rather than by genotype might be more cost-effective. However, there will be scenarios where the marginal and interactions effects for a given locus are not correlated, and where this is true the index variants may not have phenotypic proxies that could be used to recall on. Moreover, even where such proxy phenotypes exist, the relationship between them and the outcomes of the trial may not be causal, which may introduce confounding.

In some cases, the costs of genotyping large sampling-frames may be a rate-limiting factor; for instance, where a single GBR validation trial will be performed and a sampling frame dedicated to that study must be generated. In that setting, the cost-benefit ratio of a GBR trial may well be unfavorable. However, our paper is set against the backdrop of several very large bioresources that have been (or will soon be) extensively genotyped. The generation of these sampling frames and the high costs of genotyping the entire sample is justified on the basis that the sampling frame will be utilized for many different scientific objectives.

In summary, we have performed simulations to demonstrate the statistical power characteristics of GBR trials, showing ubiquitously that this is a more powerful design than conventional recruitment strategies when testing genotype × treatment interactions. To facilitate the design of future GBR trials, we have incorporated our work into a web interface for calculating power in GBR trials (https://gbr-power.crc.med.lu.se).

## Additional Information

**How to cite this article**: Atabaki-Pasdar, N. *et al*. Statistical power considerations in genotype-based recall randomized controlled trials. *Sci. Rep.* **6**, 37307; doi: 10.1038/srep37307 (2016).

**Publisher’s note:** Springer Nature remains neutral with regard to jurisdictional claims in published maps and institutional affiliations.

## Supplementary Material

Supplementary Figures and Tables
