# Supplementary material for: Statistical power considerations in genotype-based recall randomized controlled trials
Source: Sci Rep. 2016 Nov 25;6:37307. doi: 10.1038/srep37307 (PMC5122840; doi:10.1038/srep37307)
Supplement: Supplementary Figures and Tables [file srep37307-s1.pdf]

**Statistical power considerations in genotype-based recall randomized controlled trials**

Naeimeh Atabaki-Pasdar, MSc[1], Mattias Ohlsson, PhD[2], Dmitry Shungin, DDS PhD[1], Azra Kurbasic, PhD[1], Erik Ingelsson, MD PhD[3], Ewan R Pearson, FRCP PhD[4], Ashfaq Ali, PhD[1,¶], Paul W. Franks, PhD[1,5,6,¶,*]

[1]Department of Clinical Sciences, Genetic and Molecular Epidemiology Unit, Lund University, Malmö, SE-205 02, Sweden

[2] Department of Astronomy and Theoretical Physics, Computational Biology and Biological Physics Unit, Lund University, Lund, Sweden

[3] Department of Medical Sciences, Molecular Epidemiology, Uppsala University, Uppsala, Sweden

[4] Division of Cardiovascular & Diabetes Medicine, Medical Research Institute, University of Dundee, Dundee, UK

[5] Department of Public Health & Clinical Medicine, Umeå University, Umeå, Sweden

[6] Department of Nutrition, Harvard School of Public Health, Boston, MA, USA.

¶These authors contributed equally to this work

*Corresponding author

Address: Department of Clinical Sciences, Genetic and Molecular Epidemiology Unit, CRC, Building 91, Level 10, Jan Waldenströms gata 35, Skåne University Hospital Malmö, Lund University, SE-205 02, Malmö, Sweden

Tel: +4640391149; Fax: +4640391222;

E-mail: Paul.Franks@med.lu.se

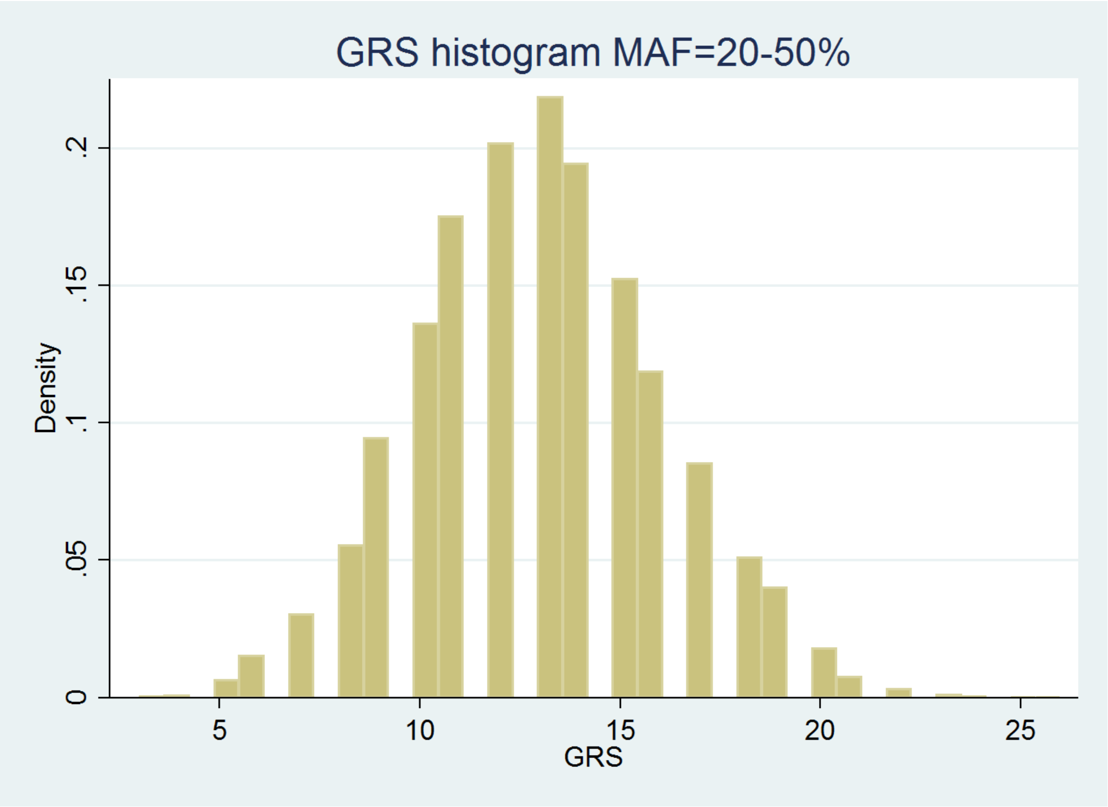


Supplementary Figure 1: Histogram of a GRS effect allele frequency distribution for 20 uncorrelated, randomly selected SNPs (minor allele frequency of 0.20-0.50) in 6,064 participants from GLACIER Study cohort

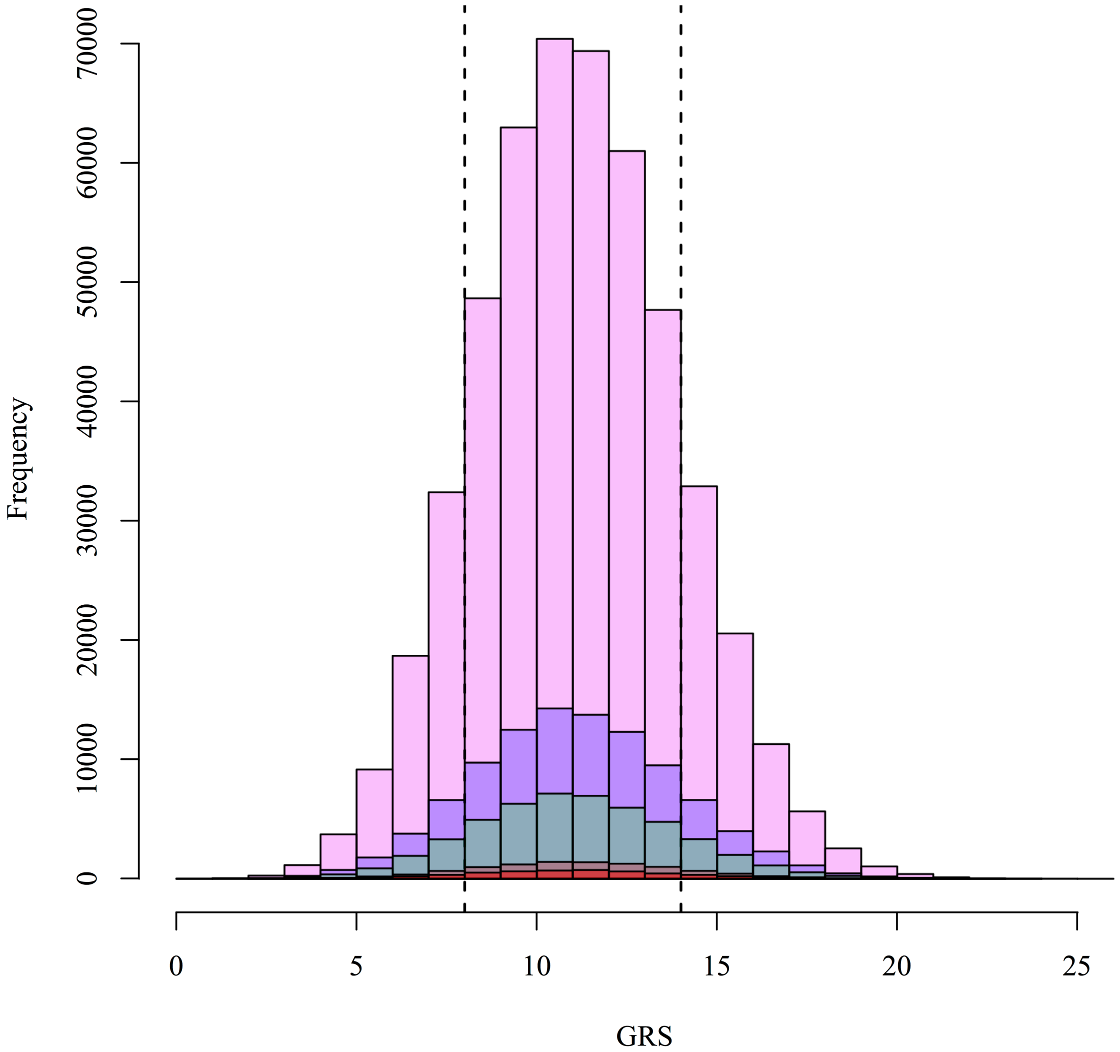


Supplementary Figure 2: Histograms of simulated GRS effect allele frequency distributions for different initial sampling-frames. The x axis indicates the GRS values and the y axis indicates allele frequencies.

| | Figure 1 | Figure 2-A | Figure 2-B | Figure 2-C | Figure 2-D |
|---|---|---|---|---|---|
| **Significance Level** | 0.05 | 0.05 | 0.05 | 0.05 | 0.05 |
| **Treatment Frequency** | 0.5 | 0.5 | 0.5 | 0.5 | 0.5 |
| **Effect Size for Treatment** | -0.5 | -0.5 | -0.5 | -0.5 | -0.5 |
| **Lowest Sample Size** | 500 | 300 | 200 | 200 | 500 |
| **Highest Sample Size** | 3500 | 700 | 2600 | 1200 | 5000 |
| **Sample integer Size** | 500 | 50 | 300 | 100 | 500 |
| **Population Sample Frame** | 31000 | 10000 | 10000 | 10000 | 10000 |
| **Interaction Effect Size for SNPs** | -0.4 | 0.17,0.30,0.22, 0.27,0.21,0.23, 0.18,0.17,0.27, 0.18,0.21,0.15, 0.28,0.28,0.30, 0.30,0.24,0.19, 0.25,0.23 | 0.06,0.11,0.06, 0.06,0.07,0.11, 0.05,0.08,0.05, 0.11,0.08,0.13, 0.07,0.13,0.07, 0.10,0.10,0.05, 0.07,0.13 | 0.17,0.30,0.22, 0.27,0.21,0.23, 0.18,0.17,0.27, 0.18,0.21,0.15, 0.28,0.28,0.30, 0.30,0.24,0.19, 0.25,0.23 | 0.06,0.11,0.06, 0.06,0.07,0.11, 0.05,0.08,0.05, 0.11,0.08,0.13, 0.07,0.13,0.07, 0.10,0.10,0.05, 0.07,0.13 |
| **Main Effect size for SNPs** | 1 | 0.46,0.34,0.40, 0.42,0.46,0.13, 0.33,0.33,0.48, 0.18,0.29,0.11, 0.42,0.25,0.33, 0.20,0.40,0.38, 0.40,0.42 | 0.34,0.10,0.25, 0.39,0.27,0.19, 0.46,0.33,0.49, 0.16,0.31,0.40, 0.49,0.26,0.26, 0.35,0.38,0.38, 0.16,0.46 | 0.46,0.34,0.40, 0.42,0.46,0.13, 0.33,0.33,0.48, 0.18,0.29,0.11, 0.42,0.25,0.33, 0.20,0.40,0.38, 0.40,0.42 | 0.34,0.10,0.25, 0.39,0.27,0.19, 0.46,0.33,0.49, 0.16,0.31,0.40, 0.49,0.26,0.26, 0.35,0.38,0.38, 0.16,0.46 |
| **Minor Allele Frequencies of SNPs** | 0.44 | 0.43,0.39,0.27, 0.37,0.27,0.28, 0.49,0.46,0.28, 0.37,0.47,0.23, 0.36,0.27,0.38, 0.42,0.40,0.25, 0.27,0.22 | 0.43,0.39,0.27, 0.37,0.27,0.28, 0.49,0.46,0.28, 0.37,0.47,0.23, 0.36,0.27,0.38, 0.42,0.40,0.25, 0.27,0.22 | 0.16,0.15,0.05, 0.20,0.13,0.13, 0.11,0.16,0.10, 0.18,0.16,0.13, 0.07,0.09,0.10, 0.15,0.15,0.20, 0.10,0.15 | 0.16,0.15,0.05, 0.20,0.13,0.13, 0.11,0.16,0.10, 0.18,0.16,0.13, 0.07,0.09,0.10, 0.15,0.15,0.20, 0.10,0.15 |
| **Type of survival time distributions** | Weibull | Gompertz | Gompertz | Gompertz | Gompertz |
| **Mean for Time to Event** | 2.165 | 2.165 | 2.165 | 2.165 | 2.165 |
| **Variance for Time to Event** | 1.24 | 1.242 | 1.242 | 1.242 | 1.24 |
| **End point for the study** | 4 | 4 | 4 | 4 | 4 |
| **Frequency of Event** | 0.376 | 0.16 | 0.16 | 0.16 | 0.16 |

Supplementary table 1: Parameters used in the simulations shown in the Figure1 and Figure 2 for Cox Regression Model (SNP).

| | Figure 3 |
|---|---|
| **Significance Level** | 0.05 |
| **Treatment Frequency** | 0.5 |
| **Effect Size for Treatment** | -1.84 |
| **Lowest Sample Size** | 200 |
| **Highest Sample Size** | 2000 |
| **Sample integer Size** | 200 |
| **Population Sample Frame** | 31000 |
| **Value for Intercept** | 767 |
| **Residual Standard Error** | 0.75 |
| **Interaction Effect Size for GRS** | 0.03 |
| **Main Effect size for GRS** | 0.66 |
| **Mean for GRS** | 36.9 |
| **Standard deviation for GRS** | 3.22 |

Supplementary table 2: Parameters used in the simulations shown in the Figure3 for Linear Regression Model (GRS).

| | Figure 4-A | Figure 4-B | Figure 4-C | Figure 4-D |
|---|---|---|---|---|
| **Significance Level** | 0.05 | 0.05 | 0.05 | 0.05 |
| **Treatment Frequency** | 0.5 | 0.5 | 0.5 | 0.5 |
| **Effect Size for Treatment** | -0.2 | -0.2 | -0.2 | -0.2 |
| **Lowest Sample Size** | 50 | 400 | 50 | 400 |
| **Highest Sample Size** | 500 | 2000 | 500 | 2000 |
| **Sample integer Size** | 50 | 200 | 50 | 200 |
| **Population Sample Frame** | 10000 | 10000 | 10000 | 10000 |
| **Interaction Effect Size for SNPs** | 0.20,0.26,0.26, 0.25,0.20,0.25, 0.21,0.27,0.16, 0.23,0.30,0.15, 0.20,0.17,0.15, 0.30,0.29,0.29, 0.25,0.23 | 0.06,0.08,0.07, 0.08,0.05,0.13, 0.06,0.13,0.06, 0.14,0.12,0.12, 0.15,0.08,0.10, 0.12,0.10,0.09, 0.09,0.07 | 0.20,0.26,0.26, 0.25,0.20,0.25, 0.21,0.27,0.16, 0.23,0.30,0.15, 0.20,0.17,0.15, 0.30,0.29,0.29, 0.25,0.23 | 0.06,0.08,0.07, 0.08,0.05,0.13, 0.06,0.13,0.06, 0.14,0.12,0.12, 0.15,0.08,0.10, 0.12,0.10,0.09, 0.09,0.07 |
| **Main Effect size for SNPs** | 0.20,0.23,0.29, 0.33,0.33,0.32, 0.35,0.23,0.24, 0.29,0.33,0.24, 0.20,0.31,0.32, 0.21,0.24,0.31, 0.28,0.25 | 0.09,0.18,0.09, 0.07,0.18,0.17, 0.12,0.17,0.08, 0.08,0.15,0.06, 0.18,0.14,0.15, 0.07,0.07,0.13, 0.06,0.08 | 0.20,0.23,0.29, 0.33,0.33,0.32, 0.35,0.23,0.24, 0.29,0.33,0.24, 0.20,0.31,0.32, 0.21,0.24,0.31, 0.28,0.25 | 0.09,0.18,0.09, 0.07,0.18,0.17, 0.12,0.17,0.08, 0.08,0.15,0.06, 0.18,0.14,0.15, 0.07,0.07,0.13, 0.06,0.08 |
| **Minor Allele Frequencies of SNPs** | 0.50,0.28,0.36, 0.43,0.48,0.47, 0.43, 0.29, 0.30, 0.20, 0.46, 0.50, 0.24, 0.29, 0.33, 0.36, 0.49, 0.46, 0.23, 0.29 | 0.50,0.28,0.36, 0.43,0.48,0.47, 0.43, 0.29, 0.30, 0.20, 0.46, 0.50, 0.24, 0.29, 0.33, 0.36, 0.49, 0.46, 0.23, 0.29 | 0.06,0.19,0.06, 0.13,0.06,0.17, 0.20,0.12,0.05, 0.15,0.16,0.16, 0.08,0.12,0.19, 0.20,0.17,0.08, 0.06,0.15 | 0.06,0.19,0.06, 0.13,0.06,0.17, 0.20,0.12,0.05, 0.15,0.16,0.16, 0.08,0.12,0.19, 0.20,0.17,0.08, 0.06,0.15 |
| **Value for Intercept** | 1 | 1 | 1 | 1 |
| **Residual Standard Error** | 3.47 | 3.47 | 3.47 | 3.47 |

Supplementary table 3: Parameters used in the simulations shown in the Figure4 for Linear Regression Model (SNP)

| | Figure 5-AB Red | Figure 5-AB Orange | Figure 5-AB Green | Figure 5-AB Blue | Figure 5-AB Purple |
|---|---|---|---|---|---|
| **Significance Level** | 0.05 | 0.05 | 0.05 | 0.05 | 0.05 |
| **Treatment Frequency** | 0.5 | 0.5 | 0.5 | 0.5 | 0.5 |
| **Effect Size for Treatment** | -0.5 | -0.5 | -0.5 | -0.5 | -0.5 |
| **Lowest Sample Size** | 200 | 200 | 200 | 200 | 200 |
| **Highest Sample Size** | 2200 | 2200 | 2200 | 2200 | 2200 |
| **Sample integer Size** | 200 | 200 | 200 | 200 | 200 |
| **Population Sample Frame** | 5000 | 10000 | 50000 | 100000 | 500000 |
| **Interaction Effect Size for SNPs** | 0.06,0.09,0.06, 0.13,0.05,0.13, 0.08,0.10,0.08, 0.09,0.13,0.12, 0.08,0.14,0.08, 0.07,0.06,0.07, 0.11,0.05 | 0.06,0.09,0.06, 0.13,0.05,0.13, 0.08,0.10,0.08, 0.09,0.13,0.12, 0.08,0.14,0.08, 0.07,0.06,0.07, 0.11,0.05 | 0.06,0.09,0.06, 0.13,0.05,0.13, 0.08,0.10,0.08, 0.09,0.13,0.12, 0.08,0.14,0.08, 0.07,0.06,0.07, 0.11,0.05 | 0.06,0.09,0.06, 0.13,0.05,0.13, 0.08,0.10,0.08, 0.09,0.13,0.12, 0.08,0.14,0.08, 0.07,0.06,0.07, 0.11,0.05 | 0.06,0.09,0.06, 0.13,0.05,0.13, 0.08,0.10,0.08, 0.09,0.13,0.12, 0.08,0.14,0.08, 0.07,0.06,0.07, 0.11,0.05 |
| **Main Effect size for SNPs** | 0.24,0.42,0.42, 0.39,0.26,0.10, 0.47,0.44,0.36, 0.18,0.35,0.39, 0.46,0.49,0.24, 0.37,0.39,0.17, 0.46,0.35 | 0.24,0.42,0.42, 0.39,0.26,0.10, 0.47,0.44,0.36, 0.18,0.35,0.39, 0.46,0.49,0.24, 0.37,0.39,0.17, 0.46,0.35 | 0.24,0.42,0.42, 0.39,0.26,0.10, 0.47,0.44,0.36, 0.18,0.35,0.39, 0.46,0.49,0.24, 0.37,0.39,0.17, 0.46,0.35 | 0.24,0.42,0.42, 0.39,0.26,0.10, 0.47,0.44,0.36, 0.18,0.35,0.39, 0.46,0.49,0.24, 0.37,0.39,0.17, 0.46,0.35 | 0.24,0.42,0.42, 0.39,0.26,0.10, 0.47,0.44,0.36, 0.18,0.35,0.39, 0.46,0.49,0.24, 0.37,0.39,0.17, 0.46,0.35 |
| **Minor Allele Frequencies of SNPs** | 0.06,0.19,0.06, 0.13,0.06,0.17, 0.20,0.12,0.05, 0.15,0.16,0.16, 0.08,0.12,0.19, 0.20,0.17,0.08, 0.06,0.15 | 0.06,0.19,0.06, 0.13,0.06,0.17, 0.20,0.12,0.05, 0.15,0.16,0.16, 0.08,0.12,0.19, 0.20,0.17,0.08, 0.06,0.15 | 0.06,0.19,0.06, 0.13,0.06,0.17, 0.20,0.12,0.05, 0.15,0.16,0.16, 0.08,0.12,0.19, 0.20,0.17,0.08, 0.06,0.15 | 0.06,0.19,0.06, 0.13,0.06,0.17, 0.20,0.12,0.05, 0.15,0.16,0.16, 0.08,0.12,0.19, 0.20,0.17,0.08, 0.06,0.15 | 0.06,0.19,0.06, 0.13,0.06,0.17, 0.20,0.12,0.05, 0.15,0.16,0.16, 0.08,0.12,0.19, 0.20,0.17,0.08, 0.06,0.15 |
| **Type of survival time distributions** | Gompertz | Gompertz | Gompertz | Gompertz | Gompertz |
| **Mean for Time to Event** | 2.165 | 2.165 | 2.165 | 2.165 | 2.165 |
| **Variance for Time to Event** | 1.242 | 1.242 | 1.242 | 1.24 | 1.24 |
| **End point for the study** | 4 | 4 | 4 | 4 | 4 |
| **Frequency of Event** | 0.16 | 0.16 | 0.16 | 0.16 | 0.16 |

Supplementary table 4: Parameters used in the simulations shown in the Figure5-AB for Cox Regression Model (SNP)

| | Figure 5-CD Red | Figure 5-CD Orange | Figure 5-CD Green | Figure 5-CD Blue | Figure 5-CD Purple |
|---|---|---|---|---|---|
| **Significance Level** | 0.05 | 0.05 | 0.05 | 0.05 | 0.05 |
| **Treatment Frequency** | 0.5 | 0.5 | 0.5 | 0.5 | 0.5 |
| **Effect Size for Treatment** | -0.2 | -0.2 | -0.2 | -0.2 | -0.2 |
| **Lowest Sample Size** | 100 | 100 | 100 | 100 | 100 |
| **Highest Sample Size** | 1000 | 1000 | 1000 | 1000 | 1000 |
| **Sample integer Size** | 100 | 100 | 100 | 100 | 100 |
| **Population Sample Frame** | 5000 | 10000 | 50000 | 100000 | 500000 |
| **Interaction Effect Size for SNPs** | 0.06,0.08,0.07, 0.08,0.05,0.13, 0.06,0.13,0.06, 0.14,0.12,0.12, 0.15,0.08,0.10, 0.12,0.10,0.09, 0.09,0.07 | 0.06,0.08,0.07, 0.08,0.05,0.13, 0.06,0.13,0.06, 0.14,0.12,0.12, 0.15,0.08,0.10, 0.12,0.10,0.09, 0.09,0.07 | 0.06,0.08,0.07, 0.08,0.05,0.13, 0.06,0.13,0.06, 0.14,0.12,0.12, 0.15,0.08,0.10, 0.12,0.10,0.09, 0.09,0.07 | 0.06,0.08,0.07, 0.08,0.05,0.13, 0.06,0.13,0.06, 0.14,0.12,0.12, 0.15,0.08,0.10, 0.12,0.10,0.09, 0.09,0.07 | 0.06,0.08,0.07, 0.08,0.05,0.13, 0.06,0.13,0.06, 0.14,0.12,0.12, 0.15,0.08,0.10, 0.12,0.10,0.09, 0.09,0.07 |
| **Main Effect size for SNPs** | 0.09,0.18,0.09, 0.07,0.18,0.17, 0.12,0.17,0.08, 0.08,0.15,0.06, 0.18,0.14,0.15, 0.07,0.07,0.13, 0.06,0.08 | 0.09,0.18,0.09, 0.07,0.18,0.17, 0.12,0.17,0.08, 0.08,0.15,0.06, 0.18,0.14,0.15, 0.07,0.07,0.13, 0.06,0.08 | 0.09,0.18,0.09, 0.07,0.18,0.17, 0.12,0.17,0.08, 0.08,0.15,0.06, 0.18,0.14,0.15, 0.07,0.07,0.13, 0.06,0.08 | 0.09,0.18,0.09, 0.07,0.18,0.17, 0.12,0.17,0.08, 0.08,0.15,0.06, 0.18,0.14,0.15, 0.07,0.07,0.13, 0.06,0.08 | 0.09,0.18,0.09, 0.07,0.18,0.17, 0.12,0.17,0.08, 0.08,0.15,0.06, 0.18,0.14,0.15, 0.07,0.07,0.13, 0.06,0.08 |
| **Minor Allele Frequencies of SNPs** | 0.50,0.28,0.36, 0.43,0.48,0.47, 0.43,0.29,0.30, 0.20,0.46,0.50, 0.24,0.29,0.33, 0.36,0.49,0.46, 0.23,0.29 | 0.50,0.28,0.36, 0.43,0.48,0.47, 0.43,0.29,0.30, 0.20,0.46,0.50, 0.24,0.29,0.33, 0.36,0.49,0.46, 0.23,0.29 | 0.50,0.28,0.36, 0.43,0.48,0.47, 0.43,0.29,0.30, 0.20,0.46,0.50, 0.24,0.29,0.33, 0.36,0.49,0.46, 0.23,0.29 | 0.50,0.28,0.36, 0.43,0.48,0.47, 0.43,0.29,0.30, 0.20,0.46,0.50, 0.24,0.29,0.33, 0.36,0.49,0.46, 0.23,0.29 | 0.50,0.28,0.36, 0.43,0.48,0.47, 0.43,0.29,0.30, 0.20,0.46,0.50, 0.24,0.29,0.33, 0.36,0.49,0.46, 0.23,0.29 |
| **Value for Intercept** | 1 | 1 | 1 | 1 | 1 |
| **Residual Standard Error** | 3.47 | 3.47 | 3.47 | 3.47 | 3.47 |

Supplementary table 5: Parameters used in the simulations shown in the Figure5-CD for Linear Regression Model (SNP).

| | Figure 7 Red | Figure 7 Blue | Figure 7 Green |
|---|---|---|---|
| **Significance Level** | 0.05 | 0.05 | 0.05 |
| **Treatment Frequency** | 0.5 | 0.5 | 0.5 |
| **Effect Size for Treatment** | -0.2 | -0.2 | -0.2 |
| **Lowest Sample Size** | 100 | 100 | 100 |
| **Highest Sample Size** | 1000 | 1000 | 1000 |
| **Sample integer Size** | 100 | 100 | 100 |
| **Population Sample Frame** | 10000 | 10000 | 10000 |
| **Interaction Effect Size for SNPs** | 0.06,0.08,0.07, 0.08,0.05,0.13, 0.06,0.13,0.06, 0.14,0.12,0.12, 0.15,0.08,0.10, 0.12,0.10,0.09, 0.09,0.07 | 0.06,0.08,0.07, 0.08,0.05,0.13, 0.06,0.13,0.06, 0.14,0.12,0.12, 0.15,0.08,0.10, 0.12,0.10,0.09, 0.09,0.07 | 0.06,0.08,0.07, 0.08,0.05,0.13, 0.06,0.13,0.06, 0.14,0.12,0.12, 0.15,0.08,0.10, 0.12,0.10,0.09, 0.09,0.07 |
| **Main Effect size for SNPs** | 0.09,0.18,0.09, 0.07,0.18,0.17, 0.12,0.17,0.08, 0.08,0.15,0.06, 0.18,0.14,0.15, 0.07,0.07,0.13, 0.06,0.08 | 0.09,0.18,0.09, 0.07,0.18,0.17, 0.12,0.17,0.08, 0.08,0.15,0.06, 0.18,0.14,0.15, 0.07,0.07,0.13, 0.06,0.08 | 0.09,0.18,0.09, 0.07,0.18,0.17, 0.12,0.17,0.08, 0.08,0.15,0.06, 0.18,0.14,0.15, 0.07,0.07,0.13, 0.06,0.08 |
| **Minor Allele Frequencies of SNPs** | 0.50,0.28,0.36, 0.43,0.48,0.47, 0.43,0.29,0.30, 0.20,0.46,0.50, 0.24,0.29,0.33, 0.36,0.49,0.46, 0.23,0.29 | 0.50,0.28,0.36, 0.43,0.48,0.47, 0.43,0.29,0.30, 0.20,0.46,0.50, 0.24,0.29,0.33, 0.36,0.49,0.46, 0.23,0.29 | 0.50,0.28,0.36, 0.43,0.48,0.47, 0.43,0.29,0.30, 0.20,0.46,0.50, 0.24,0.29,0.33, 0.36,0.49,0.46, 0.23,0.29 |
| **Value for Intercept** | 1 | 1 | 1 |
| **Residual Standard Error** | 2.2912 | 1.333 | 0.75 |

Supplementary table 6: Parameters used in the simulations shown in the Figure7 for Linear Regression Model (SNP).
